# Psychosocial Outcomes of Sharing a Diagnosis of Cancer with a Pediatric Patient

**DOI:** 10.3389/fped.2016.00070

**Published:** 2016-07-20

**Authors:** Haya Raz, Nili Tabak, Shulamith Kreitler

**Affiliations:** ^1^Nursing Department, Machon Tal, Jerusalem College of Technology, Jerusalem, Israel; ^2^Faculty of Nursing, School of Health Professions, Tel-Aviv University Medical School, Tel-Aviv, Israel; ^3^Psychooncology Research Center, Sheba Medical Center, School of Psychological Sciences, Tel-Aviv University, Tel-Aviv, Israel

**Keywords:** childhood cancer, information, childhood cancer survivors, quality of life, mental pain, meaning

## Abstract

**Purpose:**

This innovative pilot study was designed to provide research-based evidence on the variables to consider informing a child of his/her cancer diagnosis, so as to minimize the negative psychosocial effects of the cancer experience on survivors. The hypotheses of the study were that “good information” about cancer, will allow the child a better understanding way to cope with treatment and improve sociopsychological outcomes at adulthood.

**Methods:**

Ninety-one adult childhood cancer (CC) survivors got the questionnaires while waiting to their routine checkup at a grate CC medical center in center Israel.

**Results:**

To our surprise and not according to the hypothesis, there was a difference between children diagnosed up to 12 years of age and those diagnosed during adolescence. (Participants were divided into two groups according to their age at diagnosis: from birth to 12 years old and from age 12–18). In the group diagnosed at a younger age, those who had received “good information” were found to have better quality of life, lower mental pain, and higher mental pain tolerance than did those in the same group (diagnosed at a younger age) who received “not good information.” By contrast, in the group diagnosed during adolescence, those who had received “not good information” scored higher on these measures than did their counterparts who had received “good information.”

**Conclusion:**

Given that information conveyed to children diagnosed with cancer can have a significant impact on survivors’ quality of life, further research is needed to determine the precise information to be divulged to children at the time of diagnosis. In the meantime, extreme caution, sensitivity, and careful judgment are required.

**Clinical relevance:**

Findings of the current study and of future studies can be used to formulate clear guidelines for assessing a child’s readiness and the information to be divulged, so as to improve the quality of life of CC survivors.

## Introduction

Childhood cancer (CC) is a rare disease, yet it is the second most frequent cause of death among children (1). In parallel to the increase in incidence rates since the middle of the last century, the probability of survival has also consistently risen over the past decades and has reached the levels of 81–85% (2–5). The large and increasing number of survivors of CC has given impetus to the study of late effects of the disease and its treatments. Medical problems that are very frequent among CC survivors are high (6) and include even increased risk of early death of 7% at 30 years (7). In recent years, attention of researchers has turned also to the psychological sequelae of the diagnosis and treatment of CC.

There are many reasons to expect psychosocial effects following the disease and treatments. The purpose of the present study is to examine, most probably for the first time, the psychosocial outcomes of the information about cancer diagnosis shared with the child. The various stages of CC diagnosis and treatment are replete with ethical dilemmas; key among them is the issue of communicating with the child in a manner that provides age-appropriate information regarding the illness, its prognosis, and the treatment, while attempting to make the child a partner in the decision-making process (8). Sharing the diagnosis with the child is a critical aspect of the child’s care and a topic that has received little evidence-based exploration in the pediatric oncology literature.

At the “day-one talk” (9), an expert multidisciplinary team tell the parents/guardian about the cancer diagnosis and prognosis for their child. Then the parents and the oncologist must decide what to tell the child. In terms of the dilemma of whether any disease-related information should be shared with the child, the official guidance given to hospital staffs favors information sharing, according to the child’s age and cognitive abilities, and the oncologist’s decision (10).

In general, the process of diagnosing CC is a period of intense emotions for the family members involved; the ongoing stress that characterizes this period has a disruptive effect on parents’ ability to function, make judgments, and formulate decisions, which in turn can have a traumatic effect on the child and the parents alike (8, 11). The parents must simultaneously absorb and process the emotionally disturbing information, comprehend the explanations provided by the medical staff regarding the illness and its treatment and, in the middle of this emotional turmoil, (a) sign an informed consent form permitting treatment to begin and (b) decide whether and what to tell their child. Their response and their decisions depend to a large extent on their beliefs about health, their values, and the relationships within the family (12, 13). Ultimately, most will accept the hospital staff’s advice, which – as previously mentioned – is not rooted in EBP. Given this state of affairs, the goal of this study was to attempt to provide evidence in support of the hypothesis that sharing a cancer diagnosis with the child is beneficial in the long term.

### Sharing or Withholding Information: Potential Benefits and Risks

Regardless of whether the information related to the disease and diagnosis is intentionally relayed to pediatric patients with CC, seeing their parents in a state of evident anxiety and trauma (14) could very well upset these children’s sense of security. In view of their children’s fragile state, parents are not likely to punish or even admonish the transgression, thus unwittingly undermining children’s sense of security (15, 16).

In weighing, the option of sharing disease-related information with a child, it is important to note that a decision against sharing does not mean that the child has no access to information. In general, an individual accumulates information across many dimensions, among them smells, sounds, verbal cues, physiological changes, others’ attitudes, feelings, and emotions. Kreitler and Kreitler’s theory of Meaning Assignment in Perception (17) explains the ways in which individuals attribute meaning and ascribe significance to the world that surrounds them and to the objects (referents) and the occurrences in it. Infants and toddlers with cancer do not have the cognitive ability to understand theoretical explanations of their illness; hence, according to this theory, they interpret their world and its events based on whatever is communicated to them through their surroundings. Thus, in the absence of EBP, young patients with CC might conclude that their parents have failed to protect them from strangers (medical staff) and from unfamiliar procedures (tests, treatments, etc.). Without a proper explanation, they are liable to feel that a change has come and their parents no longer care for them, that the world and environment to which they had been accustomed has been transformed. In this scenario, the buffer system (18), which until then had constituted the child’s natural defense system against trauma, namely, the presence and functioning of the parents, is liable to be found fallible. Although there is a recognized stage at which individuals realize that their parents are fallible, and that in general, parents cannot protect one from experiencing anxiety, pain, or fear, in terms of mental health development, this should not take place at an unsuitably tender age, nor should this realization be foisted upon a child (by withholding information, as in the scenario described). In this sense, a decision not to share disease-related information with a child with CC runs the risk of severely damaging the child’s view of the world and of the self (18). When the absence of appropriate information is coupled with severe and traumatic treatments, a patient’s treatment compliance is liable to be adversely affected, both during childhood, as well as during the survivor’s follow-up periods, which in the long run could compromise efforts to treat physical and mental side effects.

However, a decision to share disease-related information with a pediatric patient with CC is not devoid of risk. Even if the information gathered from various dimensions does include a verbal explanation, the force of the accumulated information may overwhelm the child. According to Solomon’s Trauma Management Theory (18), many children conclude that if their new life circumstances are negative (e.g., painful treatments) this must have been caused by something negative within them and, hence, it is a form of punishment which they deserve (16).

In tow studies (19, 20), parents of children 0–7 years old who were treated for cancer, and their nurses, were asked to describe the children’s needs to feel more secure. Among the results, the sixth need was “honest communication,” and the seventh was “information.” Bachanas et al. (21) and Claflin (22) compared school age children with cancer and HIV and concluded that those who had not been given information about their diagnosis were more vulnerable and unprotected from anxiety and worries. No harm was found in children that were given information, on the contrary, the communication with them was open and helpful (23).

Whatever the child’s age at diagnosis, inevitably, the overall impression of the experience will be traumatic and connected with the way the parents coped during the difficult period (11). Talking with the child about the disease, the circumstances and the emotions it entails are ways of inviting the child to recognize the difficulties and to join the adults in the efforts to cope with the situation. This type of approach is said to help in building resilience, and there is evidence in the literature demonstrating an association between the development of resilience and positive emotional outcomes related to CC (24).

Adolescent patients with CC are at a critical stage in developing an understanding of their environment; they begin confronting existential issues, and become increasingly aware of their own mortality. According to Orbach (25) in Becker (26), healthy adolescents debate the issue of existence internally and actively choose life. However, a life-threatening illness at this stage of their emotional development could turn into a source of anxiety, which is liable to deteriorate into a severe depression (27, 28) and Post-Traumatic Stress (29).

Childhood cancer survivors gradually begin to interpret this episode in their past, while they continue to accrue new and more mature data and insights (17). Their experience of the illness and the information provided to them at the time will inevitably affect their physical and psychosocial health later in life (30).

### Purpose of the Study

In a previous phase of this project (30), it was shown that CC survivors suffer from mental pain and that it affects their quality of life. In the present phase of the project, we focused on the relations of mental pain and quality of life to variables representing the information imparted to the patients. The purpose of the study was to examine and compare the relationships between adult CC survivors’ demographics, the type of information (good/not good) shared with them upon CC diagnosis, and the psychosocial outcomes (meaning survivors attribute to CC experience, mental pain, mental pain tolerance, and quality of life).

## Materials and Methods

### Sample and Setting

Criteria for inclusion in the sample: CC survivors, who had been under continuous follow-up care in a large medical center in central Israel, were currently 18 years of age or older, had displayed no physical or laboratory evidence of cancer recurrence since completing their treatment protocol, and were proficient in Hebrew. During the data collection period (January 2009 – May 2010), 236 CC survivors matched the eligibility criteria; of these, 122 agreed to participate. Thirty-one CC survivors were later excluded, because they either failed to complete the questionnaire or refused to sign an informed consent form. This left a total of 91 participants (i.e., a final response rate of 38%). There are known difficulties in recruitments of CC survivors for studies (31–33), mainly because they would like to avoid recall of their past pain, fears, and suffering and minimize the chances that the memory of the painful experiences may be revived. The response rate in studies of this population in the literature is as low as 49 and 43.6% as reported by Rosoff et al. (34), 47% by McClellan et al. (35), and 47.5% by Casagranda et al. (36) and Gianinazzi et al. (37). Response rates as low as 45% or even 39% were obtained in some studies dealing with topics of quality of life or health, respectively [(38), p. 4], which resemble the themes in the present study.

### Data Collection Procedure

A convenience sampling method was used to recruit survivors at their routine follow-up visit. The first author approached patients in the waiting room of a large medical center in central Israel and asked them to anonymously complete a self-report questionnaire and sign an informed consent form. The study was approved by the institutional ethics committee.

### Measures

The independent variable in the study was the psychosocial well-being of adult CC survivors. The following criteria were considered measures of psychosocial well-being: (a) past and present mental pain (MP); (b) MP tolerance; (c) quality of life (QoL); and (d) the meaning of CC.

The dependent variables:

*Mental pain* (a): we studied MP in CC survivors for two main reasons: (a) studies show discrepancies and unambiguous findings of late psychosocial effects of CC. For example: low QOL (39) and high QOL (40) and so on for behavioral issues that most of the studies focused on. (b) The evidence of prevalence suicide and suicidal attempts in CC survivors (41–44). Considerations of these reasons led to study mental pain for the first time in this population. Mental pain is a construct whose role in regards to suicide has been emphasized from the very beginning (45, 46). Its concerns are “how much you hurt as a human being.” Mental pain is considered as a unique subjective experience, different from depression and anxiety with which it was found to share some cognitions (46).

Mental pain was measured using the Orbach and Mikulincer Mental Pain Scale (46), which measures past MP (at diagnosis) and present MP. The questionnaire comprises 43 items grouped according to the nine characteristics of MP: (1) Loss of control, e.g., “I lack control over what is happening inside me”; (2) Irreversibility of the pain, e.g., “Something in my life changed forever”; (3) Emotional flooding, e.g., “There is a storm of emotions in me”; (4) Narcissistic wounds, e.g., “No one is interested in me”; (5) Estrangement, e.g., “It is as if I were not real”; (6) Confusion, e.g., “I cannot concentrate”; (7) Need for social support, e.g., “I need support from my surroundings”; (8) Emptiness, e.g., “I have no desire for anything”; (9) Freezing, e.g., “It’s like I’m paralyzed.” Respondents were asked to rate each statement on a scale, from 1 – *Strongly disagree* to 5 – *Strongly agree*, according to the extent to which the statement described their own MP. The questionnaire had been validated in studies of suicide (47). The internal reliability scores in the current study were Cronbach α = 0.96 for current mental pain and α = 0.97 for past mental pain [validation by Guimara et al. (48)].

In the present study, the internal reliability scores were 0.96 Cronbach’s alpha for present MP and 0.97 Cronbach’s alpha for past MP.

Mental pain *tolerance (b)*: The mental pain tolerance section of the questionnaire consisted of 20 items, each rated on the same five-point Likert scale options as the scale of mental pain at present. It provides scores on the three following scales: (1) Congestion – the extent to which the pain occupies a person, the extent to which the person can ignore the pain and concentrate on other things (e.g., “I just cannot stand the pain”); (2) Coping – the long-term capacity to manage the pain, so that when a person feels there is no hope for the pain to stop, and that they cannot act to obtain relief, then the pain experience can be unbearable and devastating (e.g., “I can do nothing to reduce the pain”); and (3) Containment – refers to feeling the pain without having to secure immediate relief in any way possible, including impulsive behaviors, such as attempted suicide (e.g., “I feel I have to get rid of the pain immediately”). A higher score on mental pain tolerance reflected a higher capacity for mental pain tolerance. This section of the questionnaire was validated in studies of suicide (46). Its internal reliability in the current study was α = 0.96.

*Quality of life (c)*: The *Quality of Life* questionnaire used in the study was developed by Kreitler and Kreitler (49) and validated in studies of cancer survivors of different ages (50). It is currently also used to evaluate the QoL of CC survivors in Israeli follow-up clinics. The questionnaire comprises 62 questions answered by checking one of four options, ranging from 1 (the most positive) to 4 (the most negative). The questions span a variety of daily functioning components, including family relationships; leisure-time activity; work activity; negative feelings; positive feelings; cognitive function; physical function; social activity and social relationships; life-quality image; sense of mastery; self-esteem; motivation; stress; and basic needs. The measurement scale for QoL was reversed so that a low score indicated low quality – and a high score indicated a high QoL. The questionnaire’s internal reliability score in the current study was 0.96 Cronbach’s alpha.

*Meaning attributed to the CC experience (d)*: Section 1 of Kreitler’s structured meaning questionnaire (17) was used to evaluate the participants’ perception of their CC experience. Section 1 examined the meaning attributed to the CC experience by presenting nine statements related to common experiences of CC (cancer, oncology, chemotherapy, nurse, doctor, hospital, treatments, tests, and leukemia/lymphoma) and respondents were asked to describe in their own words what these concepts and representations meant to them (e.g., Treatments: “treatments can cause pain and nausea, but can save your life”). A panel of judges analyzed the replies, coding them on a scale ranging from 1 (very negative) to 5 (very positive); disagreements were discussed until a consensus was reached. Finally, the total score for the nine statements was assigned either a positive or a negative value, indicating the meaning attributed to the CC experience overall.

*The independent variable*: The main dependent variable in the present study was the quality of the information the child received about the cancer diagnosis and has three dimensions or components: (a) content, (b) timing, and (c) source.

Section 2 of Kreitler’s structured meaning questionnaire (17), was adapted so to obtain data regarding the diagnosis-related information the survivor was given as a child. Respondents are presented with 31 open-ended questions about how they came to be informed of their CC (e.g., “did the medical staff talk to you about the disease? If so, what were you told? What words did the staff use? What do you remember your parents telling you about the disease?”). The internal reliability score for Section 2 of the questionnaire in the present study was 0.91 Cronbach’s alpha. Also the descriptive replies to this section of the questionnaire were analyzed and coded by a panel of judges. In 95% of instances, the panel was unanimous in its categorization of the respondents’ answers.

*Information content (a)*: This has five components: (a) The name used to refer to the illness, *for example*, “I remember the word *oncology* not *cancer*”; “They used the word *illness* not *cancer*”; (b) the description or explanation of the illness as relayed to the child, the manner in which it was pictured or exemplified, e.g., “an invasion of bad cells,” “killing off,” or “destroying” the bad cells; (c) The description of the treatments. Most of the metaphors used were borrowed from the context of war and defeating an enemy, i.e., the illness; (d) the description of the expected side effects: weakness, tiredness, vomiting, hair loss; (e) the prognosis delivery.

*The timing of the delivery of information (b)*: Timing the delivery of information is critical for children’s understanding of the course of treatment. As noted earlier, depending on their age and cognitive ability, children may expect parents and significant others to protect and shield them. Hence, the focus of the timing component was on whether the child was informed at the time of diagnosis, which was measured using a dichotomous yes/no item, whereby no = 1 and yes = 2.

*The information source (c)*: The staff members of CC units are highly experienced at helping parents plan and present the disclosure of the diagnosis to the child. The underlying assumption, for which we seek EBP in this study, is that a well-planned disclosure of information provided near the time of the actual diagnosis will be beneficial to the adult CC survivor’s long-term well-being. In contrast, it is assumed that children, who are given the information “incidentally” and without preparation, are liable to form an inaccurate idea of their own particular diagnosis, sometimes one even worse than the true situation. Therefore, the source of information was defined as either a preplanned discussion facilitated or assisted by a medical team member vs. incidental information delivery. Replies were scaled dichotomously as follows: was the child given information? (no = 1, yes = 2); when was the information given? (1 = at diagnosis, 2 = not at diagnosis); and what was the source of the information? (1 = medical team member\present in – a preplanned discussion and 2 = incidental information).

Finally, based on the scores on all of the three subsections that constitute the information variable, a final score (on a scale from 1 to 5) was assigned to the entire information quality variable. Accordingly, it was determined that a score of 3 or above 3 would be considered *good information* (GI), and a score below 3 would be considered *not good information* (NGI). The statistical analyses conducted henceforth involved comparisons between the resulting two groups of the sample: the group of participants who received GI and the group of participants who received NGI.

The issue of retrospective recall of past experiences regarding CC diagnosis and treatments deserves clarification. It needs to be emphasized that the authors do not assume that recall at present represents the past experience in any way. Assessing the present recall of the cancer experience was designed merely to examine the manner in which the survivors represent to themselves the past events. These representations are important insofar as they constitute a component in the narrative that the survivors construct of their past and forms a part of the conceptual and emotional complex of memories, emotions, and attitudes concerning their biography and anamnesis with which they cope and on the basis of which they construct at present their future life narrative (51). Memories of past are an essential part of one’s autobiographical memory were shown to contribute to one’s self identity and help in the process of making sense of the past for the purpose of improved quality of life (52, 53). Hence, the main independent variable is the perception of the information given to the CC survivor.

The following hypothesis was posited: participants who had received GI (a score of 3 or above on Section 2 of the questionnaire) during their CC episode would exhibit more positive psychosocial conditions (MP, QoL, meaning of CC) than would their counterparts who had been given NGI at the time of their illness.

## Results

### Sample Characteristics

The 91 participant sample was characterized by a fairly even distribution in terms of gender (50 men and 41 women) and types of childhood diagnosis: lymphoma – 41 (45%), leukemia – 21 (23%), and other diagnoses – 29 (32%). Population-sector distribution was as follows: 92% of the respondents were native Israelis and 8% had immigrated to Israel; 2% (*n* = 2) were Arab-Israelis, and the rest were Jewish; in terms of religious affiliation, 78% (*n* = 71) described themselves as secular Jews, 19% (*n* = 17) as religious Jews; and on one questionnaire, the question of religious affiliation was ignored. Most of the participants (71%, *n* = 65) were unmarried. The mean age at diagnosis was 12 years (range: 1–23 years), median 11.50 years, and mean current age was 26 years (range: 18–43 years), median 25.00 years. Mean time elapsed since the end of treatment was 13 years (range: 1–38 years), median 13.00 years.

The normality of the data distributions was tested by *Z* tests for skewness and kurtosis. On all variables, the scores were found to be distributed in the desired range of ±1.96. Meaning: The sample’s overall mean score for the meaning of CC was 3.31 (of 5; SD = 0.54); in other words, most of the survivors attributed a moderately positive meaning to their CC experience. The sample was divided into two groups, as previously described: the GI group consisted of 49 participants (54%) and the NGI group consisted of 42 participants (46%). Statistically significant differences were found between the two information groups in terms of age at diagnosis and time since the end of treatment (see Table 1), but not in terms of present age. Table 2 shows the two groups’ distribution by gender and by diagnosis.

**Table 1:** **Information groups by age at diagnosis, present age, and time since end of treatment (means and SD)**.

Groups	Good information		Not good information		

Factors	M	SD	M	SD	t
Age at diagnosis	9.55	5.44	12.82	4.78	3.05**
Present age	25.54	5.69	25.55	6.10	0.01
Time since end of treatment	14.59	6.88	11.35	7.11	2.21*

*p < 0.05.

**p < 0.01.

**Table 2:** **Information groups by gender and diagnosis (*N* = 91)**.

	Groups	Good information	Not good information			*X*^2^

Factors		*N*	%	*N*	%	
Gender	Men	23	54.8	27	55.1	0.00
	Women	19	45.2	22	44.9	
Diagnosis	Leukemia	18	42.9	23	46.9	0.43
	Lymphoma	11	26.2	10	20.4	
	Other	13	31.0	16	32.7	

**Figure 1:**
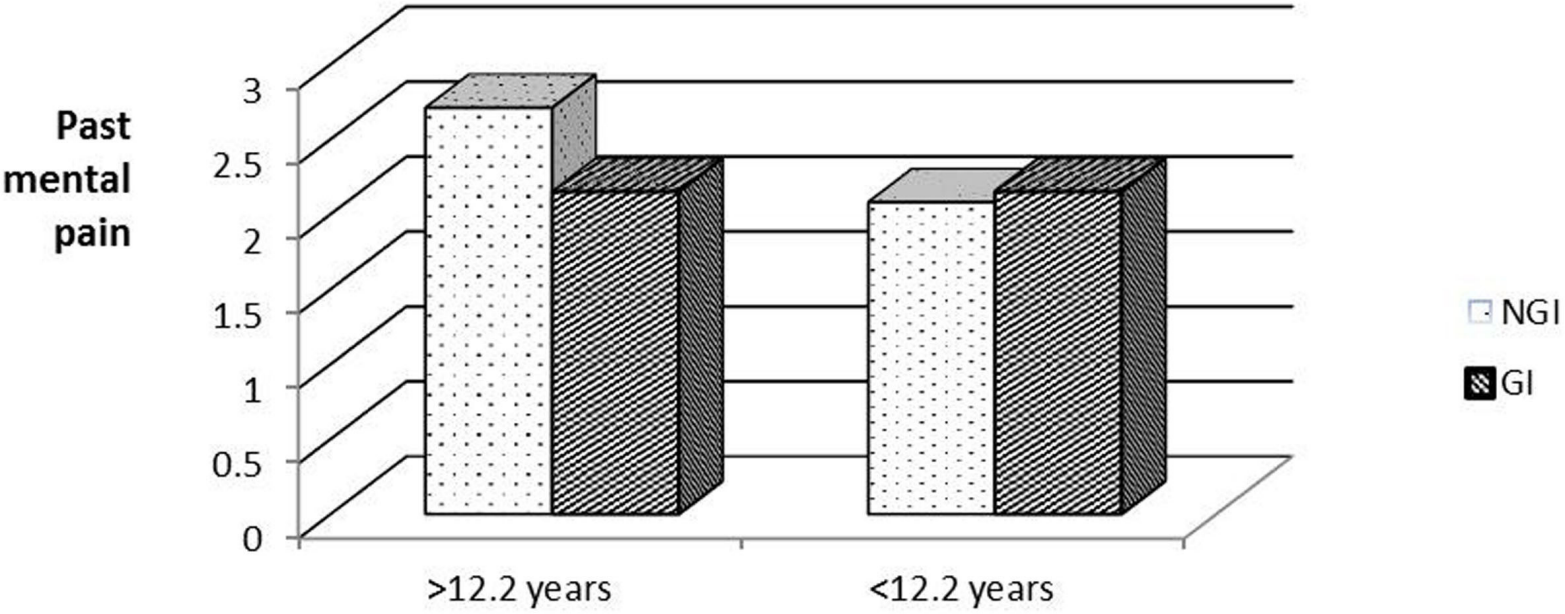
**Past mental pain by age at diagnosis groups and information groups**.

To examine whether age at diagnosis had an effect on subsequent (past and present) psychosocial well-being, the respondents were divided into two groups by age at diagnosis (below and above the median age of 12.2 years). Figure 1 shows a statistically significant interaction between age at diagnosis and past MP [*F*(1,86) = 8.26, Eta^2^ = 0.09]. In the younger group, those who received NGI reported higher levels of past MP than did those who received GI. Surprisingly, in the older age group, those who received GI reported higher past MP than those who received NGI.

A Simple Effects analysis was conducted to test for the source of the above interaction, while comparing the GI/NGI groups in each age group separately. A significant difference was found between the GI/NGI groups in the younger age group [*F*(1,40) = 6.35, *p* < 0.05, Eta^2^ = 0.12], but not in the older age group [*F*(1,40) = 2.64, *p* > 0.05]. Surprisingly, members of the older age group who received GI reported higher past MP than those who received NGI.

Pearson correlations were calculated between the information groups and the variables of past and present MP, MP tolerance, QoL, and the meaning attributed to the CC experience (see Table 3).

**Table 3:** **Pearson correlation coefficients between groups by dependent variables**.

	Quality of life	Present mental pain	Past mental pain	Mental pain tolerance	Childhood cancer meaning
Childhood cancer meaning	0.487***	**−0.231**	**0.112**	0.300*	
Mental pain tolerance	0.565***	−0.589***	−0.352*		0.081
Past mental pain	**−0.003**	0.316*		−0.459**	0.265*
Present mental pain	−0.686***		0.548***	−0.599***	−0.046
Quality of life		−0.707***	0.526***	0.421***	−0.007

*p < 0.05.

**p < 0.01.

***p < 0.001.

*The figures in bold represent respondents who received “good information” (*N* = 49); the non-bold figures represent respondents who received “not good information” (*N* = 41)*.

The only statistically significant difference between the GI and NGI groups identified by Fisher *Z* tests (*Z* = *Z* = 2.69, *p* < 0.01) was related to the relationship between the variables of past MP and QoL.

In the NGI group, a significant negative correlation was found between past MP and QoL; in the GI group, the correlation was weaker and did not reach statistical significance. Significant positive correlations were found in the GI group between CC meaning, MP tolerance, and QoL. By contrast, these correlations were low and non-significant in the NGI group. Fisher *Z* tests indeed found a significant inter-group difference only with respect to the correlation between CC meaning and QoL (*Z* = 2.28, *p* < 0.05). As for the association between MP tolerance and QoL, in both information groups, the two variables were positively correlated. As noted, above, a negative correlation was found in both of the information groups between past MP and QoL. However, in terms of groups’ actual rankings, in the NGI group, past MP was high and QoL was low, while opposite was the case in the GI group: past MP was low and QoL was high.

A hierarchical regression analysis was performed to measure the combined contribution of the study variables in explaining the variance in present MP, MP tolerance, and QoL. In performing the regression analysis for present MP and MP tolerance, the predictor variables were entered in five steps: (1) two sociodemographic data – age at diagnosis and gender; (2) the GI/NGI variable; (3) past MP; (4) CC meaning; and (5) the interactions between GI/NGI groups and other variables (to test whether the variables’ contribution to the explanation of variance is the same in both information groups). We also examined the interactions between gender, age at diagnosis, past MP, and CC meaning. The aim was to see if all of these variables together explained the variance better than did each variable separately. In performing the regression analysis for QoL, the first four steps were identical to those performed in the regression analysis for present MP and for MP tolerance. At the fifth step, however, present MP and MP tolerance were entered, in order to see how much these two variables added to the explanation of variance over and above the other variables. It should be noted that at every step, except for the step in which the interactions were entered, the entering of the predictor variables was forced, whereas for entering the interactions, the criterion was statistical significance. That is, only interactions whose contribution to the explanation of variance was statistically significant were entered.

The hierarchical regression analysis found that the predictor variables explained 30% of the variance in present MP and 62% of the variance in QoL. As shown in Table 4, age at diagnosis and gender (Step 1) explained 9% of the variance and gender was the only factor with a statistically significant contribution. The beta coefficient for gender is positive, meaning that present MP is higher among women than among men. At Step 2, entering the information-group variable did not reveal a statistically significant contribution. The entering of past MP at Step 3 added 17% to the explanation of variance. The beta coefficient for this variable is positive, indicating that respondents with high past MP also reported high present MP. The entering of CC meaning at Step 4 added a further 4% to the explanation of the variance. The beta coefficient for this variable is negative, so that respondents who reported attributing a positive meaning to the CC experience also reported low present MP.

**Table 4:** **Hierarchical regression analysis to explain variance of present mental pain (*N* = 91)**.

*B* variables	1	2	3	4
Age at diagnosis	0.061	0.086	0.089	0.103
Gender	0.299**	0.299**	0.256**	0.256**
Information group		−0.084	−0.055	−0.061
Past mental pain			0.412***	0.440***
Childhood cancer meaning				−0.191*
*R*^2^	0.09*	0.10*	0.27***	0.30***
Δ*R*^2^	0.09*	0.01	0.17***	0.03*

*p < 0.05.

**p < 0.01.

***p < 0.001.

Reviewing the findings of the regression analysis to explain the variance in QoL (Table 5) demonstrates that the personal data and information-group factors (Steps 1 and 2) did not add a statistically significant contribution to the explanation of variance. Past MP (Step 3) contributed 6.3% to explaining QoL variance. The beta coefficient for past MP is negative, indicating that higher past MP correlated with lower QoL. Meaning attributed to the CC experience (Step 4) contributed 11% to explaining QoL variance, such that the more positive the meaning attributed to CC, the higher was the QoL score assigned by participants. MP tolerance and present MP (Step 5) explained a further 36% of the QoL variance. The entering of present MP markedly reduced the beta coefficient for past MP, indicating that present MP is a mediating variable between past MP and QoL. A Sobel test confirmed the significance of this mediation (*Z* = 4.01, *p* < 0.001). Only present MP the added a statistically significant contribution to the explanation of QoL variance. Its beta coefficient was negative, so that the higher the present MP, the lower was the QoL. At Step 6, three interactions were added to the regression analysis – between information group and MP, between information group and MP tolerance, and between CC meaning and past MP. Together these three interactions contributed a further 8% to the explanation of variance so that, in all, 62% of was explained.

**Table 5:** **Hierarchical regression analysis to explain variance of quality of life (*N* = 91)**.

*B* variables	1	2	3	4	5	6
Age at diagnosis	0.028	0.034	0.032	0.008	0.078	0.012
Gender	−0.121	−0.121	−0.095	−0.095	0.079	0.122
Information		−0.018	−0.035	−0.026	−0.025	−0.011
Past mental pain			−0.254*	−0.302**	0.027	0.040
Childhood cancer meaning				0.333***	0.184*	0.163*
Past mental pain					−0.667***	−0.617***
Mental pain tolerance					0.083	0.127
Information × past mental pain						0.247***
Information × mental pain tolerance						0.207**
Meaning × present mental pain						0.158*
*R*^2^	0.02	0.02	0.08	0.19	0.54***	0.62***
Δ*R*^2^	0.02	0.00	0.60*	0.10**	0.35***	0.08***

*p < 0.05.

**p < 0.01.

***p < 0.001.

**Figure 2:**
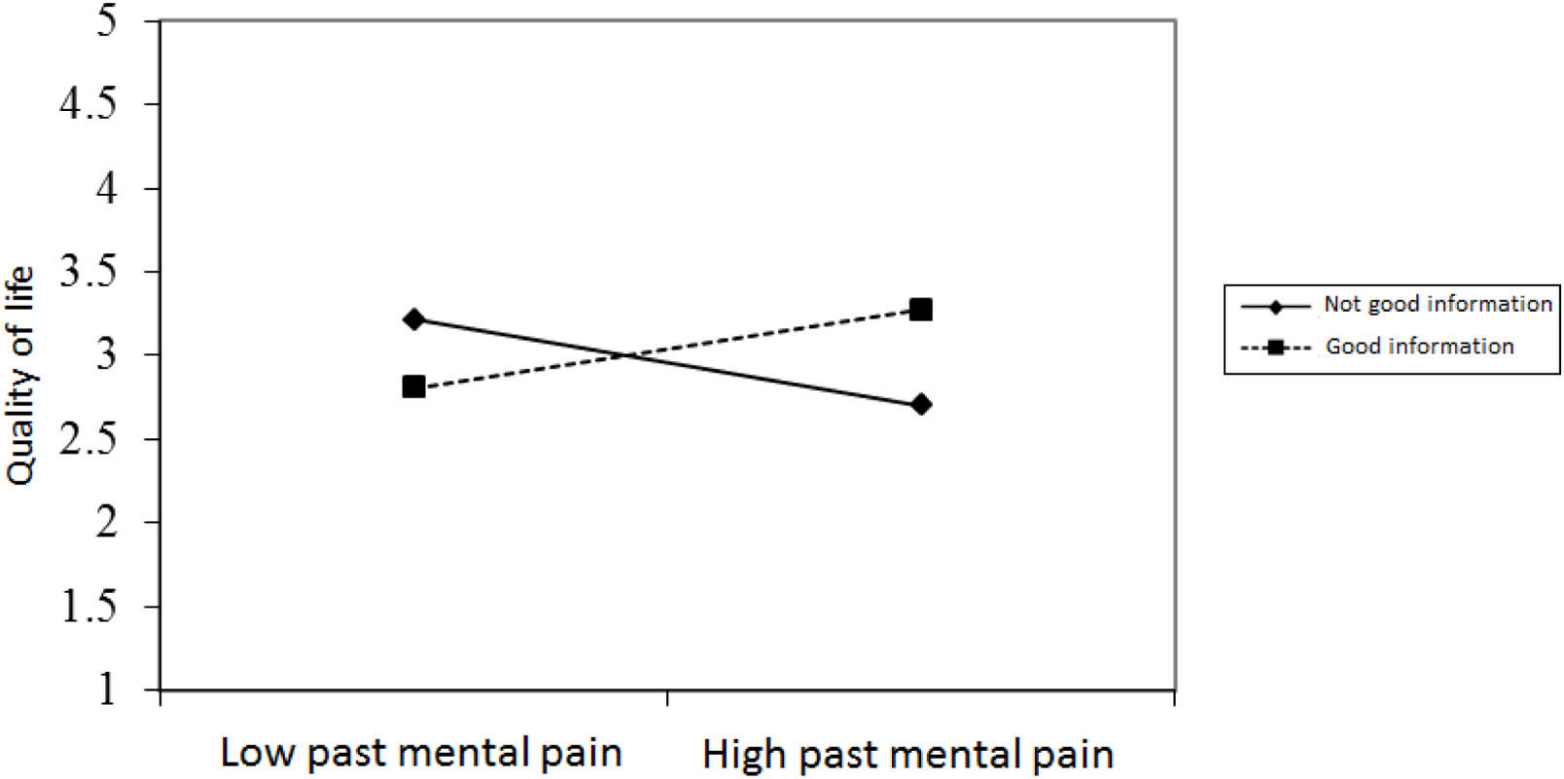
**The relationship between past mental pain and quality of life in the two information groups**.

**Figure 3:**
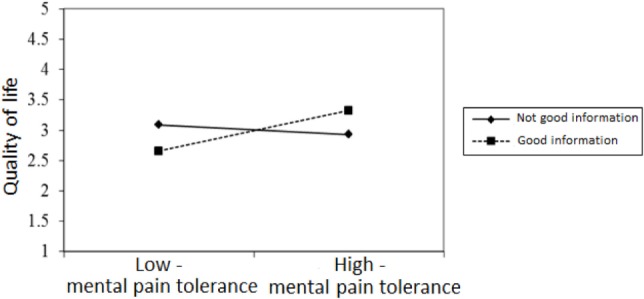
**The relationship between mental pain tolerance and quality of life in respondents who received good and not good information**.

**Figure 4:**
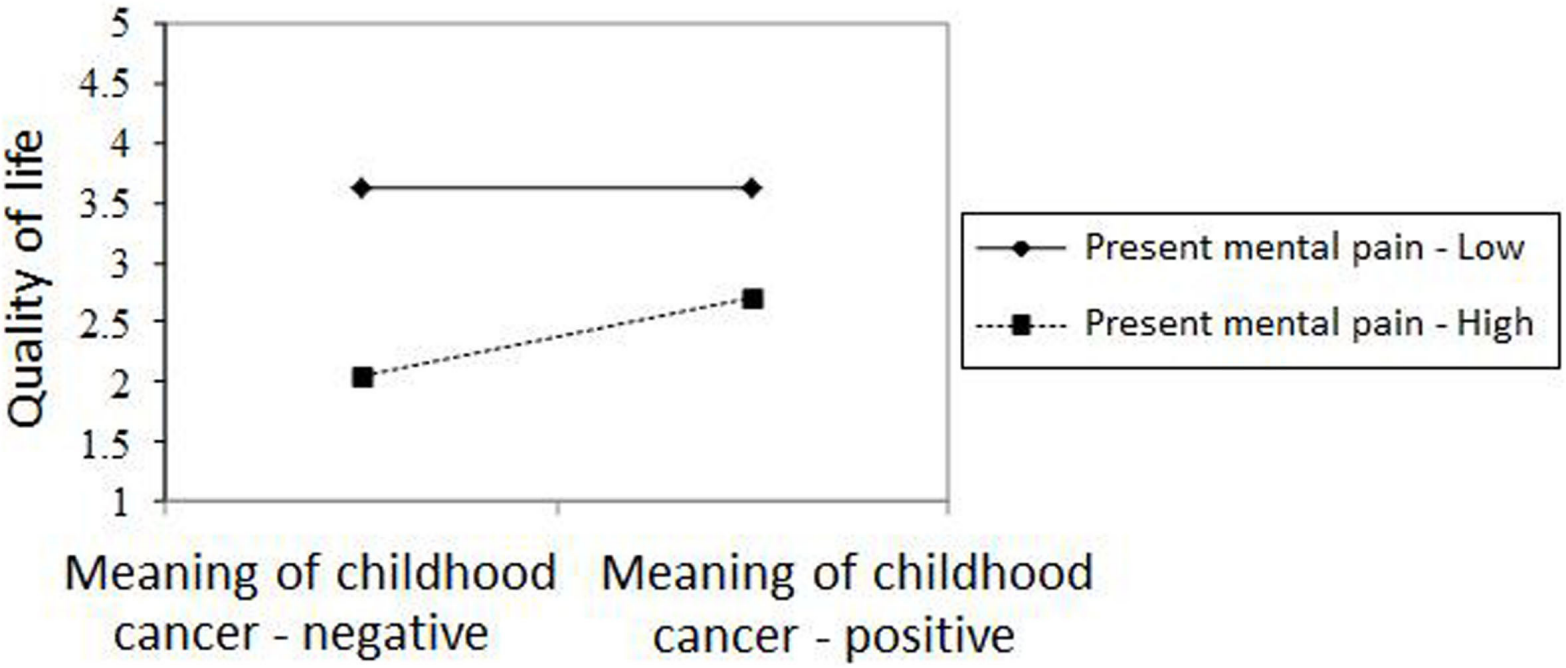
**The relationship between meaning of cancer in childhood and quality of life in respondents with high/low present mental pain**.

Figures 2–4 chart the interactions, which made significant contributions to the explanation of variance. Figure 2 shows that in the GI group, higher past MP correlated with higher scores on QoL, whereas in the NGI group, higher past MP correlated with lower scores on QoL. Figure 3 charts the statistically significant interaction between information group and MP tolerance. As for the interaction between MP and CC meaning (show in Figure 4), it seems that among respondents with low present MP, the meaning attributed to the CC experience had no effect on QoL. However, it should be noted that overall, respondents with low present MP reported a higher QoL than did respondents with higher present MP. Correspondingly, among respondents who reported high present MP, the more positive the meaning they attributed to the CC experience, the higher were their reported QoL levels.

## Discussion

The findings of this initial study underscore the need to examine this important subject regarding information provided to a child diagnosed with cancer (the survivors’ perception of the information) and to research its long-term implications and effects. The study was conducted in a large medical center in Israel. The underlying assumption, which was disproved, was that nowadays this issue is no longer problematic. The sample’s overall mean score for the significance attributed to the CC experience was 3.31 (of 5; SD = 0.54), in other words, most of the survivors attributed a moderately positive meaning to their CC experience. Past MP was higher than present MP, MP tolerance scores tended to be high (3.62), and the QoL average (3.47) was also considered good.

### Differences between the Information Groups

Interestingly, the two information groups (GI group/NGI group) into which the sample was divided were similar in size as well as in most demographic features. In the current study, the respondents’ age at diagnosis, and the time elapsed since then differed significantly between the two information groups. By contrast, there was no difference found between the groups in terms of the type of cancer diagnosed. In the NGI group, the mean age at diagnosis was 13 years compared to a mean age of 10 years in the GI group. According to the predominant claim in the literature, the older the child is and, consequently, the more cognitively advanced the child is, the greater the likelihood that sharing information about diagnosis and treatment will have a positive long-term effect (9, 54). The current finding does not uphold this claim. It is possible that the discrepancy between this finding and the current understanding promoted in the professional literature can be explained by the practices upheld by the physicians that do not follow the advice in the literature and/or the way to share this information with the child, is to be studied further for enabling the survivors memories of the past improve their QOL.

As noted, the two sample groups (GI/NGI) were not differentiated by the type and severity of their cancer despite the fact that the different types of cancer differ widely by prognosis and survival rate. It may be assumed that in the case of patients with cancer types associated with a relatively good prognosis, staff would be more prone to speak with the child regarding the disease, or at least less likely to avoid the subject entirely (55–57). The finding of our study, namely the absence of any significant effect related to the type of cancer, contradicts this assumption. It seems that it is difficult to talk about cancer with a child, regardless of the particular diagnosis or prognosis.

### Differences between the Age Groups

A significant difference between the information groups was found in relation to participants’ age at diagnosis. *According to the findings, CC* survivors who were under 12 years of age when told of their diagnosis and who received GI attributed – as adults – a more positive meaning to their CC experience, and the psychosocial effects of that experience were also less severe (less MP, higher MP tolerance, higher QoL) than were those of survivors diagnosed in the same period (younger than 12 years old) who received NGI. This may be explained by assuming that young children are at a cognitive stage that does not enable them to grasp the severity of the disease. Hence, an explanation that can lead them to understand their condition can come only later in the course of their lives, when they are already survivors. Over the years, survivors continue to take in information about their illness from a variety of sources, and their perception of their cancer experience changes and develop accordingly (17).

The two information groups did differ significantly on the variable of time since end of treatment (*t* = 2.21, *p* < 0.05), but once again this finding ran counter to expectations. More time had elapsed (15 years on average) in the GI group than in the NGI group (12 years on average). Given that therapies and survival rates for CC have improved considerably in the last 20 years and that information about cancer is so much more readily accessible, we expected that the group with the better psychosocial outcomes would also be the one more recently diagnosed, thus substantiating the beneficial effects of more recent knowledge – not only knowledge about the disease but also knowledge about how to talk to patients about it. After all, these improvements have made it easier for staff to talk to the pediatric patient with greater optimism for the future. However, as noted, this expectation was refuted.

The respondents’ descriptions of the information given them, as children, about their diagnosis make it abundantly obvious that frequently the information given is of a highly problematic sort. The following quotes provide examples that suggest that the information that was provided did not have the desired effect. “Only much later, around the age of 18, did I for the first time realize what my illness had been”; “They tried their hardest to lie to me”; “I couldn’t talk about it because everyone evaded answering or didn’t know how to answer my questions.” Many respondents reported that one of the hardest things they remembered was their parents’ grief: “… seeing my parents in tears, I don’t remember much else …”: “My mother was weeping all the time and they told me that it was a serious illness, called cancer.” Despite efforts to the contrary, including Israel’s Patients’ Rights Act of 1996, and proven improvements in therapies, survival rates, supportive care for side effects, and accessibility of information about cancer on the Internet and elsewhere, the adverse long-term psychosocial effects of a CC diagnosis have not been curbed. Most importantly, the existence of clear recommendations in the professional literature regarding the manner in which disease-related information should be presented to a young patient with CC has not helped minimize long-term traumatic effects of CC on the psychosocial well-being of the CC survivor. Nevertheless, findings suggest that GI is a critical factor in survivors’ quality of life; hence, we must aspire to continue our efforts to ensure survivors’ psychosocial, as well as physical, health. This topic has not been sufficiently researched and current practices are not supported by research-based evidence. We are convinced that this line of research should be further pursued and that, on the basis of its findings, oncological staff should be trained to provide “good information” to a child diagnosed with cancer.

### Limitations of the Study

The study’s sample size was small for dividing it to two groups by age at diagnosis. This was a pilot study and for more significant results a bigger sample size is needed. As we wrote above, the childhood cancer survivors population is a very sensitive one, and there are ethical issues involved with asking them to participate in a study. The study is a retrospective study that asked the participants to answer according to their recollection. A prospective study will have more accuracy.

### Recommendations

Every decision, procedure, and/or action taken by medical staff in the matter of giving information or withholding information from a child diagnosed with cancer ought to be EBP. We believe that the precise information to be provided to a child with CC at the time of diagnosis, in light consideration of the child’s age and needs, should be vigorously researched. In the meantime, before providing such information, the medical team should exercise extreme caution, care, sensitivity, and judgment, and the information should be communicated only after assessing the child’s ability to absorb and contain the diagnosis. Such heightened attention is called for because the information can have a significant impact on to CC survivors’ QoL.

We recommend for further research based on the findings in our study and questions they raise: to conduct a prospective study about sharing information according to the child’s age, provide EBP, the kind of information we should provide at adolescents and how to guide medical staff and parents to share information with their child at diagnosis and later on. This will help CC survivors in the process of making sense of the past for the purpose of improved QoL.

## Author Contributions

Each author have participated sufficiently in the work to take public responsibility for appropriate portions of the content.

## Conflict of Interest Statement

The authors declare that the research was conducted in the absence of any commercial or financial relationships that could be construed as a potential conflict of interest. The reviewers MH, JY, AB, and handling Editor declared their shared affiliation, and the handling Editor states that the process nevertheless met the standards of a fair and objective review.
